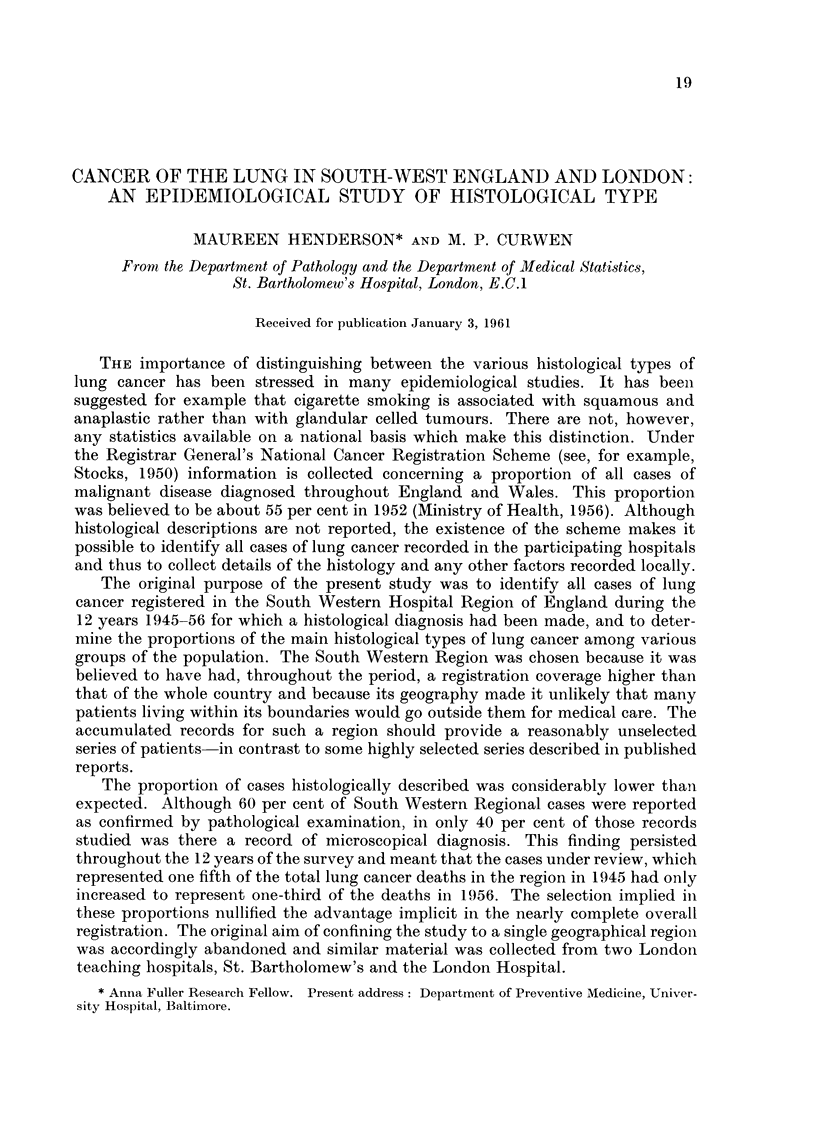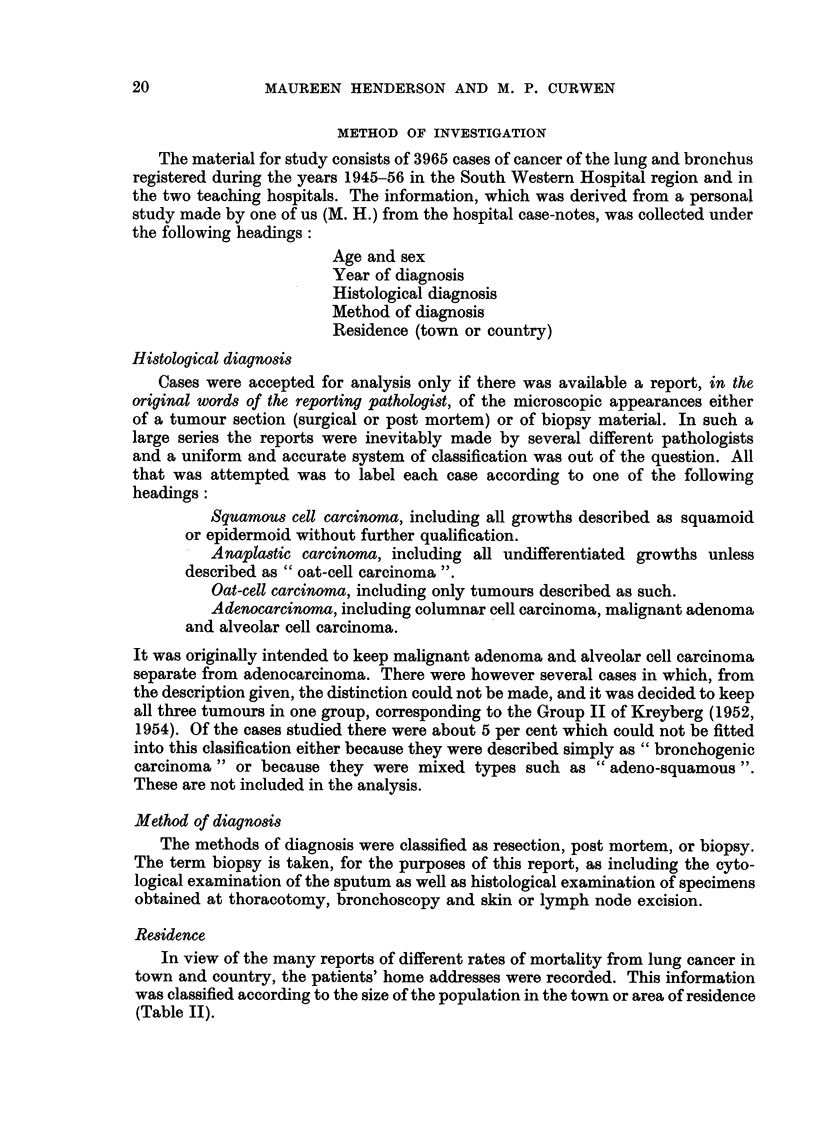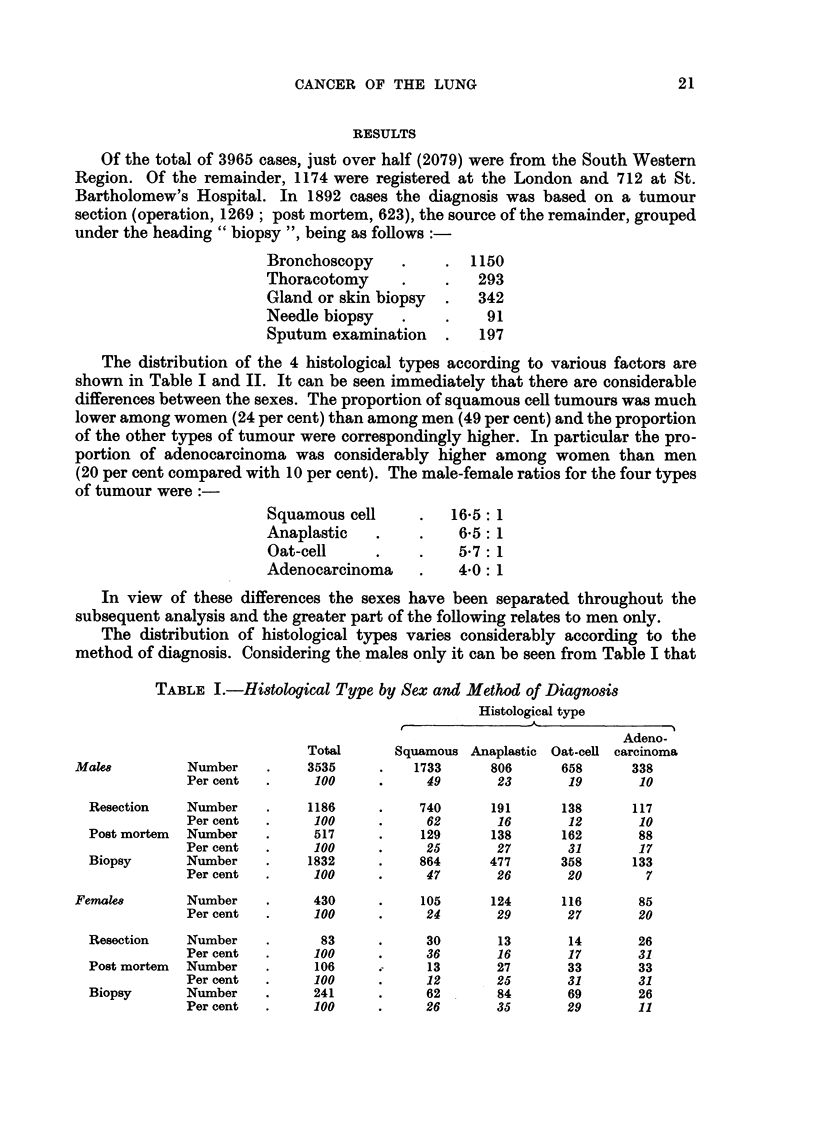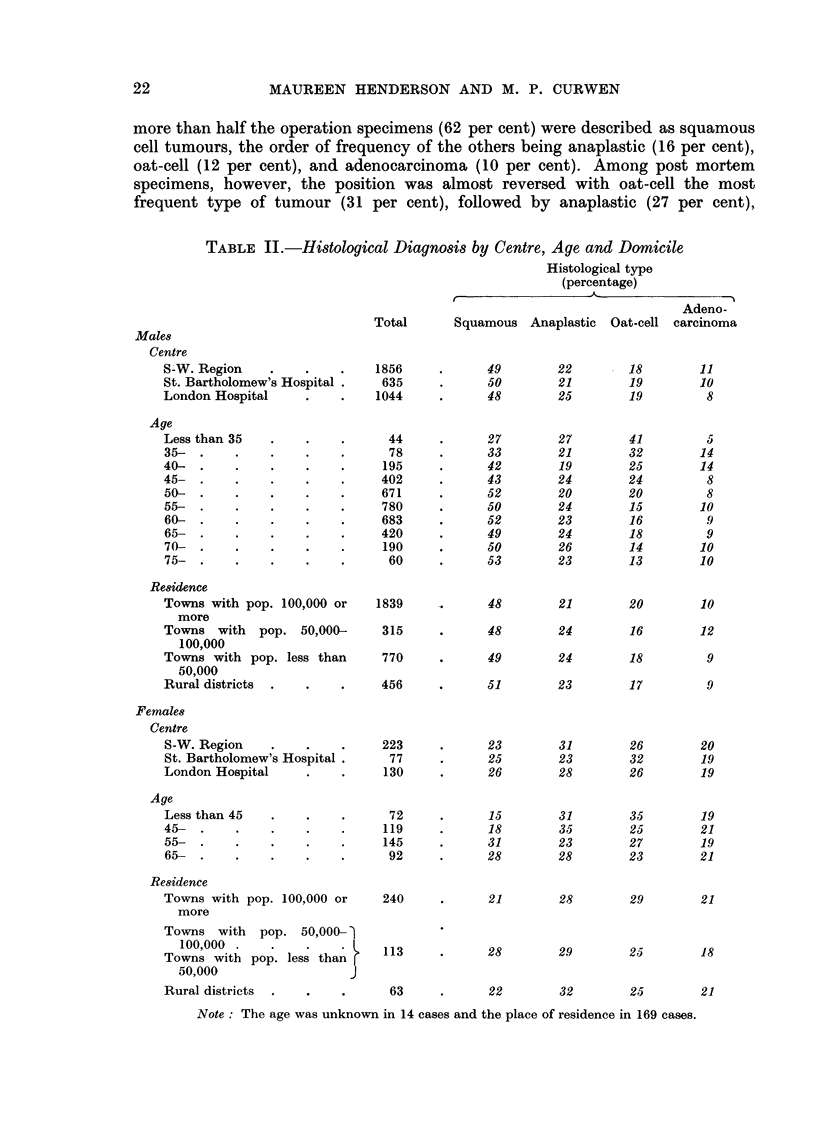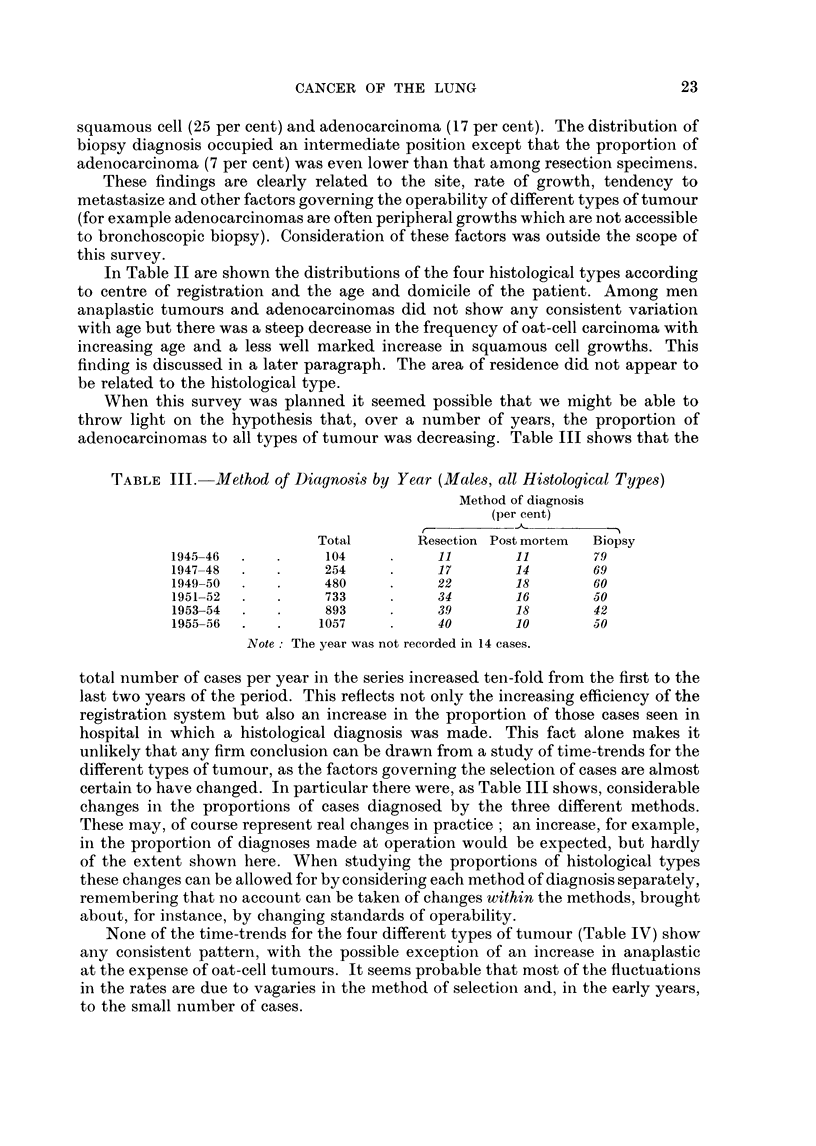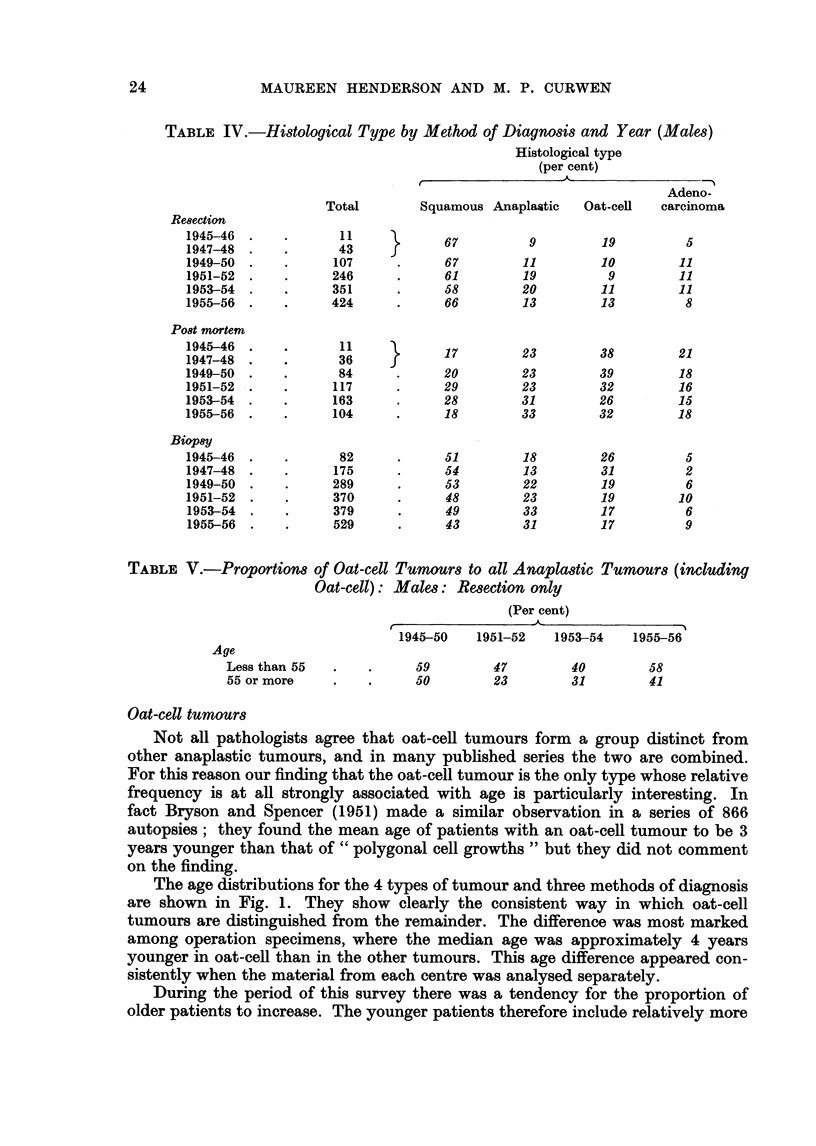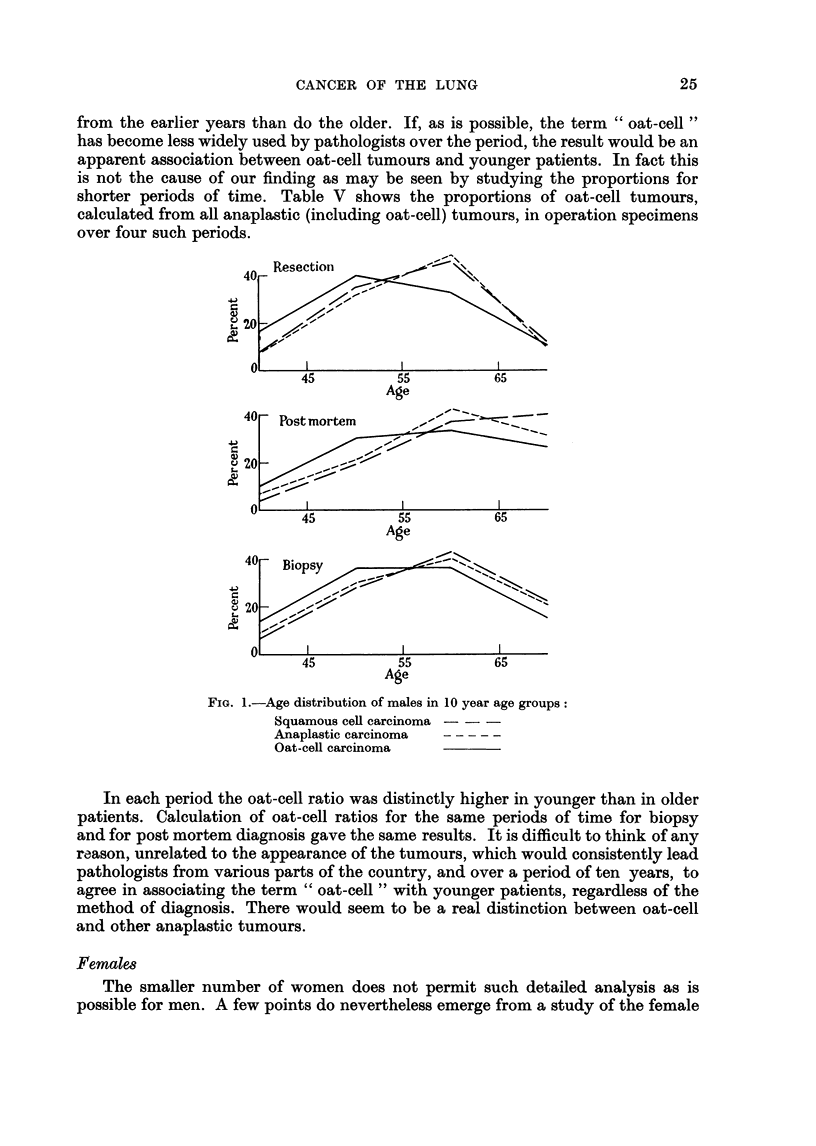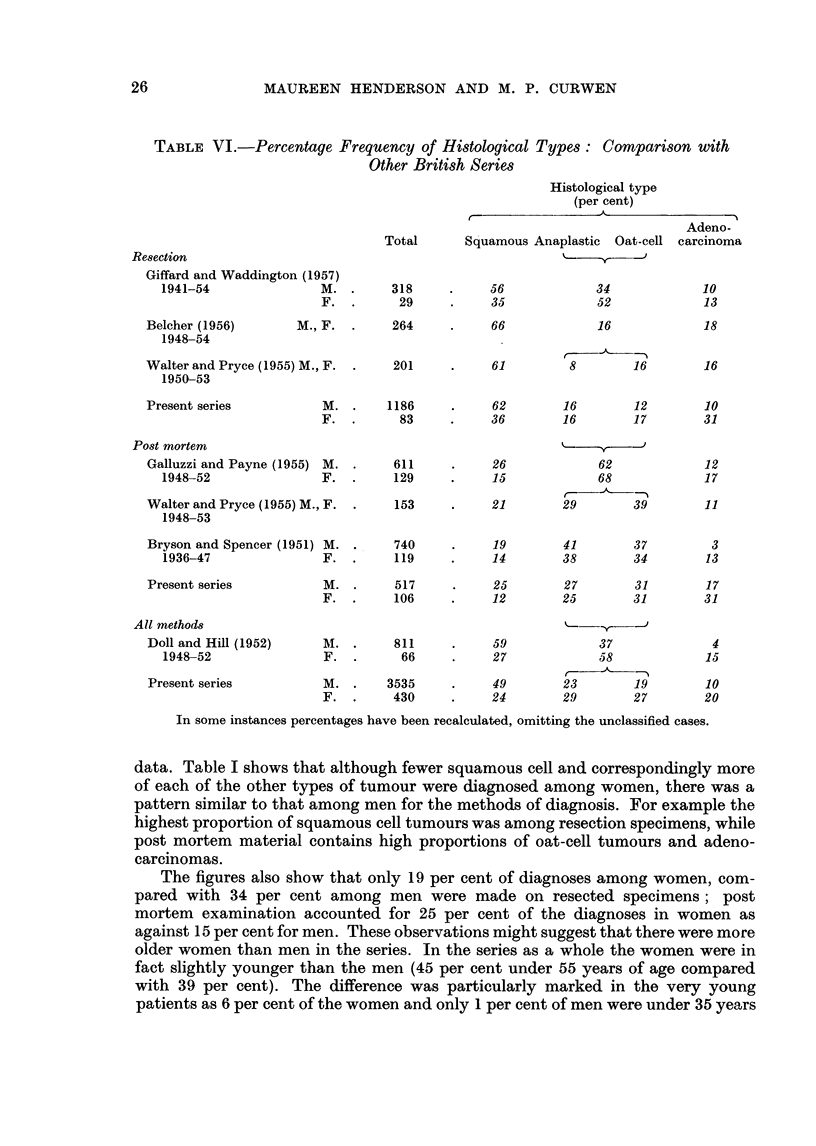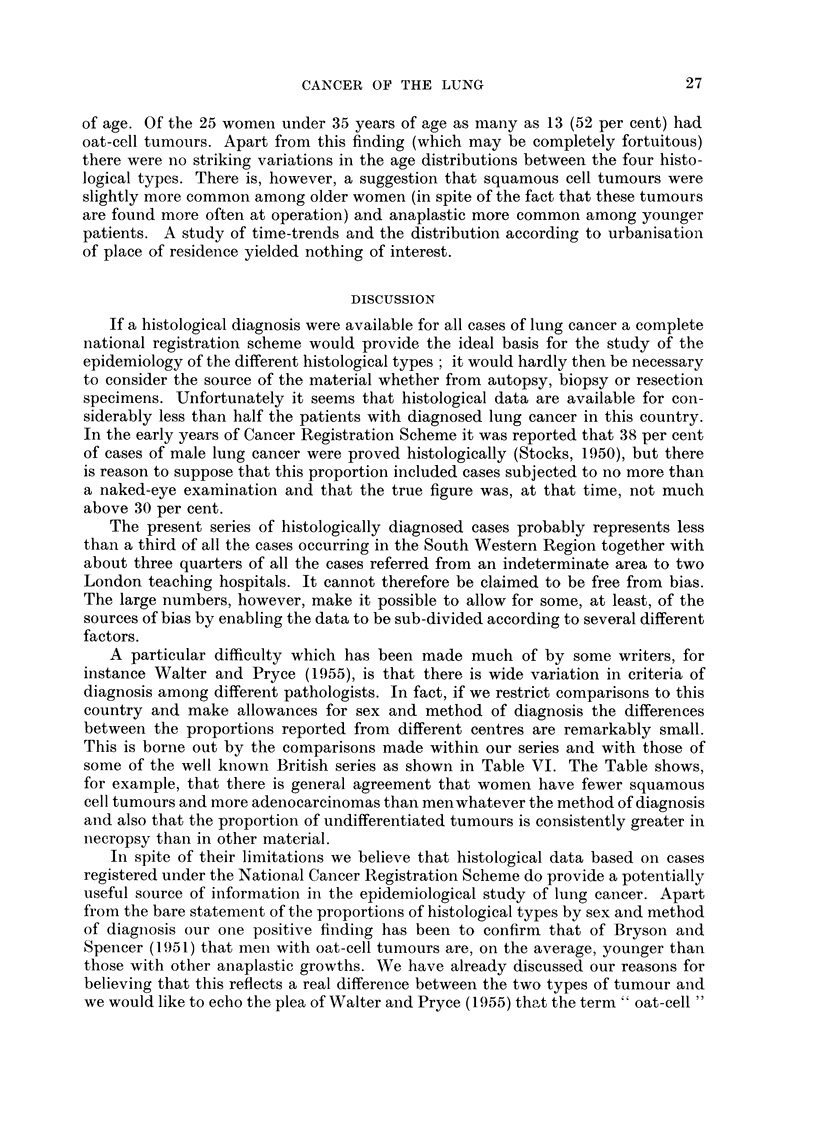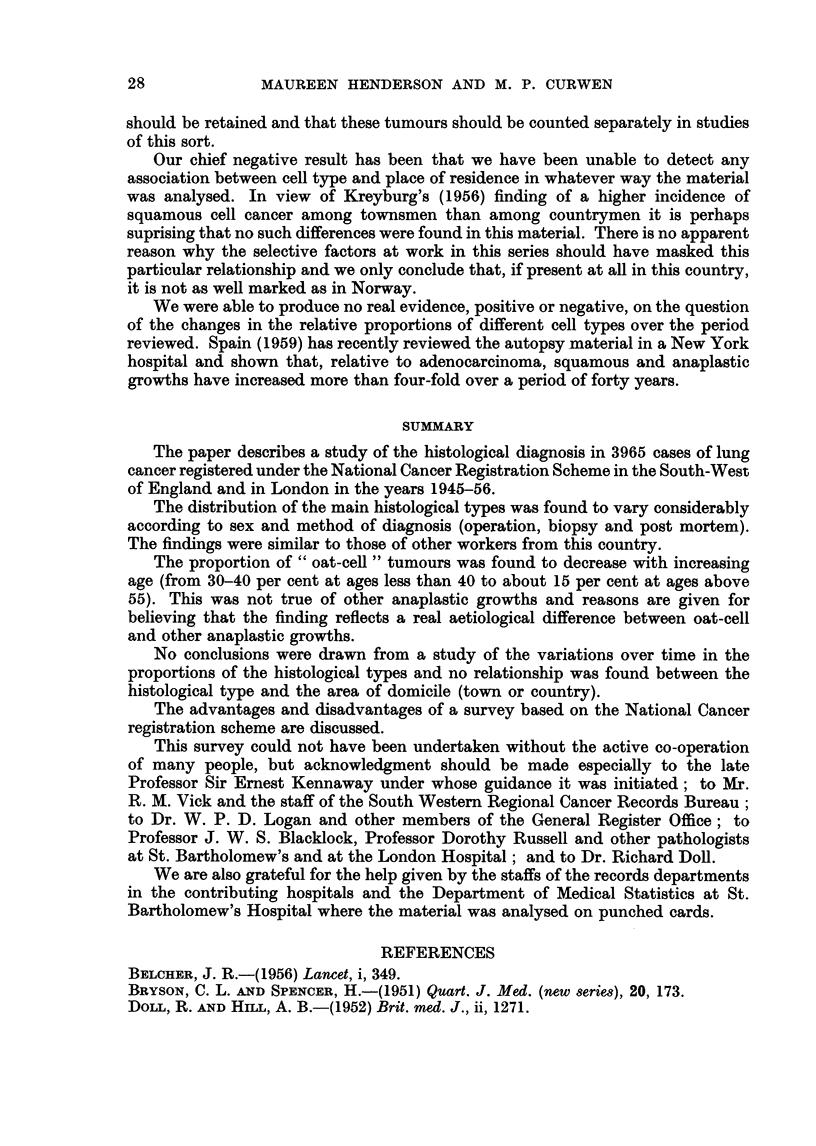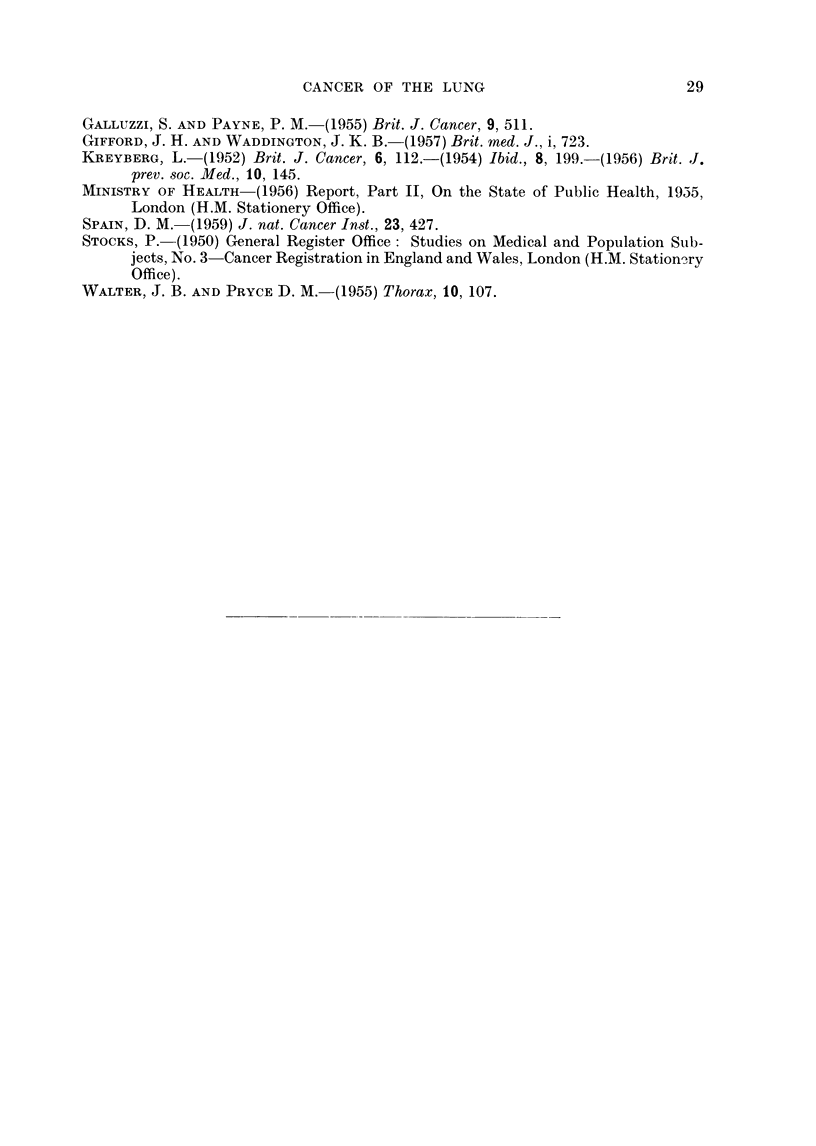# Cancer of the Lung in South-West England and London: an Epidemiological Study of Histological Type

**DOI:** 10.1038/bjc.1961.3

**Published:** 1961-03

**Authors:** Maureen Henderson, M. P. Curwen


### CANCER OF THE LUNG IN SOUTH-WEST ENGLAND AND LONDON: AN EPIDEMIOLOGICAL STUDY OF HISTOLOGICAL TYPE

MAUREEN HENDERSON* AND M. P. CURWEN

*From the Department of Pathology and the Department of Medical Statistics, St. Bartholomew's Hospital, London, E.C.1*

Received for publication January 3, 1961

THE importance of distinguishing between the various histological types of lung cancer has been stressed in many epidemiological studies. It has been suggested for example that cigarette smoking is associated with squamous and anaplastic rather than with glandular celled tumours. There are not, however, any statistics available on a national basis which make this distinction. Under the Registrar General's National Cancer Registration Scheme (see, for example, Stocks, 1950) information is collected concerning a proportion of all cases of malignant disease diagnosed throughout England and Wales. This proportion was believed to be about 55 per cent in 1952 (Ministry of Health, 1956). Although histological descriptions are not reported, the existence of the scheme makes it possible to identify all cases of lung cancer recorded in the participating hospitals and thus to collect details of the histology and any other factors recorded locally.

The original purpose of the present study was to identify all cases of lung cancer registered in the South Western Hospital Region of England during the 12 years 1945–56 for which a histological diagnosis had been made, and to determine the proportions of the main histological types of lung cancer among various groups of the population. The South Western Region was chosen because it was believed to have had, throughout the period, a registration coverage higher than that of the whole country and because its geography made it unlikely that many patients living within its boundaries would go outside them for medical care. The accumulated records for such a region should provide a reasonably unselected series of patients—in contrast to some highly selected series described in published reports.

The proportion of cases histologically described was considerably lower than expected. Although 60 per cent of South Western Regional cases were reported as confirmed by pathological examination, in only 40 per cent of those records studied was there a record of microscopical diagnosis. This finding persisted throughout the 12 years of the survey and meant that the cases under review, which represented one fifth of the total lung cancer deaths in the region in 1945 had only increased to represent one-third of the deaths in 1956. The selection implied in these proportions nullified the advantage implicit in the nearly complete overall registration. The original aim of confining the study to a single geographical region was accordingly abandoned and similar material was collected from two London teaching hospitals, St. Bartholomew's and the London Hospital.

* Anna Fuller Research Fellow. Present address: Department of Preventive Medicine, University Hospital, Baltimore.

### METHOD OF INVESTIGATION

The material for study consists of 3965 cases of cancer of the lung and bronchus registered during the years 1945–56 in the South Western Hospital region and in the two teaching hospitals. The information, which was derived from a personal study made by one of us (M. H.) from the hospital case-notes, was collected under the following headings :

Age and sex
Year of diagnosis
Histological diagnosis
Method of diagnosis
Residence (town or country)

### *Histological diagnosis*

Cases were accepted for analysis only if there was available a report, *in the original words of the reporting pathologist*, of the microscopic appearances either of a tumour section (surgical or post mortem) or of biopsy material. In such a large series the reports were inevitably made by several different pathologists and a uniform and accurate system of classification was out of the question. All that was attempted was to label each case according to one of the following headings :

*Squamous cell carcinoma*, including all growths described as squamoid or epidermoid without further qualification.

*Anaplastic carcinoma*, including all undifferentiated growths unless described as " oat-cell carcinoma ".

*Oat-cell carcinoma*, including only tumours described as such.

*Adenocarcinoma*, including columnar cell carcinoma, malignant adenoma and alveolar cell carcinoma.

It was originally intended to keep malignant adenoma and alveolar cell carcinoma separate from adenocarcinoma. There were however several cases in which, from the description given, the distinction could not be made, and it was decided to keep all three tumours in one group, corresponding to the Group II of Kreyberg (1952, 1954). Of the cases studied there were about 5 per cent which could not be fitted into this clasification either because they were described simply as " bronchogenic carcinoma " or because they were mixed types such as " adeno-squamous ". These are not included in the analysis.

### *Method of diagnosis*

The methods of diagnosis were classified as resection, post mortem, or biopsy. The term biopsy is taken, for the purposes of this report, as including the cytological examination of the sputum as well as histological examination of specimens obtained at thoracotomy, bronchoscopy and skin or lymph node excision.

### *Residence*

In view of the many reports of different rates of mortality from lung cancer in town and country, the patients' home addresses were recorded. This information was classified according to the size of the population in the town or area of residence (Table II).

### RESULTS

Of the total of 3965 cases, just over half (2079) were from the South Western Region. Of the remainder, 1174 were registered at the London and 712 at St. Bartholomew's Hospital. In 1892 cases the diagnosis was based on a tumour section (operation, 1269 ; post mortem, 623), the source of the remainder, grouped under the heading " biopsy ", being as follows :—

| | |
|---|---|
| Bronchoscopy . . | 1150 |
| Thoracotomy . . | 293 |
| Gland or skin biopsy . | 342 |
| Needle biopsy . . | 91 |
| Sputum examination . | 197 |

The distribution of the 4 histological types according to various factors are shown in Table I and II. It can be seen immediately that there are considerable differences between the sexes. The proportion of squamous cell tumours was much lower among women (24 per cent) than among men (49 per cent) and the proportion of the other types of tumour were correspondingly higher. In particular the proportion of adenocarcinoma was considerably higher among women than men (20 per cent compared with 10 per cent). The male-female ratios for the four types of tumour were :—

| | |
|---|---|
| Squamous cell . | 16·5 : 1 |
| Anaplastic . . | 6·5 : 1 |
| Oat-cell . . | 5·7 : 1 |
| Adenocarcinoma . | 4·0 : 1 |

In view of these differences the sexes have been separated throughout the subsequent analysis and the greater part of the following relates to men only.

The distribution of histological types varies considerably according to the method of diagnosis. Considering the males only it can be seen from Table I that

TABLE I.—*Histological Type by Sex and Method of Diagnosis*

| | | Total | Histological type: Squamous | Anaplastic | Oat-cell | Adeno-carcinoma |
|---|---|---|---|---|---|---|
| *Males* | Number | 3535 | 1733 | 806 | 658 | 338 |
| | Per cent | *100* | *49* | *23* | *19* | *10* |
| Resection | Number | 1186 | 740 | 191 | 138 | 117 |
| | Per cent | *100* | *62* | *16* | *12* | *10* |
| Post mortem | Number | 517 | 129 | 138 | 162 | 88 |
| | Per cent | *100* | *25* | *27* | *31* | *17* |
| Biopsy | Number | 1832 | 864 | 477 | 358 | 133 |
| | Per cent | *100* | *47* | *26* | *20* | *7* |
| *Females* | Number | 430 | 105 | 124 | 116 | 85 |
| | Per cent | *100* | *24* | *29* | *27* | *20* |
| Resection | Number | 83 | 30 | 13 | 14 | 26 |
| | Per cent | *100* | *36* | *16* | *17* | *31* |
| Post mortem | Number | 106 | 13 | 27 | 33 | 33 |
| | Per cent | *100* | *12* | *25* | *31* | *31* |
| Biopsy | Number | 241 | 62 | 84 | 69 | 26 |
| | Per cent | *100* | *26* | *35* | *29* | *11* |

more than half the operation specimens (62 per cent) were described as squamous cell tumours, the order of frequency of the others being anaplastic (16 per cent), oat-cell (12 per cent), and adenocarcinoma (10 per cent). Among post mortem specimens, however, the position was almost reversed with oat-cell the most frequent type of tumour (31 per cent), followed by anaplastic (27 per cent),

TABLE II.—*Histological Diagnosis by Centre, Age and Domicile*

| | | Histological type (percentage) | | | |
|---|---|---|---|---|---|
| | Total | Squamous | Anaplastic | Oat-cell | Adeno-carcinoma |
| *Males* | | | | | |
| *Centre* | | | | | |
| S-W. Region | 1856 | *49* | *22* | *18* | *11* |
| St. Bartholomew's Hospital | 635 | *50* | *21* | *19* | *10* |
| London Hospital | 1044 | *48* | *25* | *19* | *8* |
| *Age* | | | | | |
| Less than 35 | 44 | *27* | *27* | *41* | *5* |
| 35– | 78 | *33* | *21* | *32* | *14* |
| 40– | 195 | *42* | *19* | *25* | *14* |
| 45– | 402 | *43* | *24* | *24* | *8* |
| 50– | 671 | *52* | *20* | *20* | *8* |
| 55– | 780 | *50* | *24* | *15* | *10* |
| 60– | 683 | *52* | *23* | *16* | *9* |
| 65– | 420 | *49* | *24* | *18* | *9* |
| 70– | 190 | *50* | *26* | *14* | *10* |
| 75– | 60 | *53* | *23* | *13* | *10* |
| *Residence* | | | | | |
| Towns with pop. 100,000 or more | 1839 | *48* | *21* | *20* | *10* |
| Towns with pop. 50,000–100,000 | 315 | *48* | *24* | *16* | *12* |
| Towns with pop. less than 50,000 | 770 | *49* | *24* | *18* | *9* |
| Rural districts | 456 | *51* | *23* | *17* | *9* |
| *Females* | | | | | |
| *Centre* | | | | | |
| S-W. Region | 223 | *23* | *31* | *26* | *20* |
| St. Bartholomew's Hospital | 77 | *25* | *23* | *32* | *19* |
| London Hospital | 130 | *26* | *28* | *26* | *19* |
| *Age* | | | | | |
| Less than 45 | 72 | *15* | *31* | *35* | *19* |
| 45– | 119 | *18* | *35* | *25* | *21* |
| 55– | 145 | *31* | *23* | *27* | *19* |
| 65– | 92 | *28* | *28* | *23* | *21* |
| *Residence* | | | | | |
| Towns with pop. 100,000 or more | 240 | *21* | *28* | *29* | *21* |
| Towns with pop. 50,000–100,000; Towns with pop. less than 50,000 | 113 | *28* | *29* | *25* | *18* |
| Rural districts | 63 | *22* | *32* | *25* | *21* |

*Note :* The age was unknown in 14 cases and the place of residence in 169 cases.

squamous cell (25 per cent) and adenocarcinoma (17 per cent). The distribution of biopsy diagnosis occupied an intermediate position except that the proportion of adenocarcinoma (7 per cent) was even lower than that among resection specimens.

These findings are clearly related to the site, rate of growth, tendency to metastasize and other factors governing the operability of different types of tumour (for example adenocarcinomas are often peripheral growths which are not accessible to bronchoscopic biopsy). Consideration of these factors was outside the scope of this survey.

In Table II are shown the distributions of the four histological types according to centre of registration and the age and domicile of the patient. Among men anaplastic tumours and adenocarcinomas did not show any consistent variation with age but there was a steep decrease in the frequency of oat-cell carcinoma with increasing age and a less well marked increase in squamous cell growths. This finding is discussed in a later paragraph. The area of residence did not appear to be related to the histological type.

When this survey was planned it seemed possible that we might be able to throw light on the hypothesis that, over a number of years, the proportion of adenocarcinomas to all types of tumour was decreasing. Table III shows that the

TABLE III.—*Method of Diagnosis by Year (Males, all Histological Types)*

| | Total | Method of diagnosis (per cent) Resection | Post mortem | Biopsy |
|---|---|---|---|---|
| 1945–46 | 104 | *11* | *11* | *79* |
| 1947–48 | 254 | *17* | *14* | *69* |
| 1949–50 | 480 | *22* | *18* | *60* |
| 1951–52 | 733 | *34* | *16* | *50* |
| 1953–54 | 893 | *39* | *18* | *42* |
| 1955–56 | 1057 | *40* | *10* | *50* |

*Note :* The year was not recorded in 14 cases.

total number of cases per year in the series increased ten-fold from the first to the last two years of the period. This reflects not only the increasing efficiency of the registration system but also an increase in the proportion of those cases seen in hospital in which a histological diagnosis was made. This fact alone makes it unlikely that any firm conclusion can be drawn from a study of time-trends for the different types of tumour, as the factors governing the selection of cases are almost certain to have changed. In particular there were, as Table III shows, considerable changes in the proportions of cases diagnosed by the three different methods. These may, of course represent real changes in practice ; an increase, for example, in the proportion of diagnoses made at operation would be expected, but hardly of the extent shown here. When studying the proportions of histological types these changes can be allowed for by considering each method of diagnosis separately, remembering that no account can be taken of changes *within* the methods, brought about, for instance, by changing standards of operability.

None of the time-trends for the four different types of tumour (Table IV) show any consistent pattern, with the possible exception of an increase in anaplastic at the expense of oat-cell tumours. It seems probable that most of the fluctuations in the rates are due to vagaries in the method of selection and, in the early years, to the small number of cases.

TABLE IV.—*Histological Type by Method of Diagnosis and Year (Males)*

| | Total | Histological type (per cent) Squamous | Anaplastic | Oat-cell | Adeno-carcinoma |
|---|---|---|---|---|---|
| *Resection* | | | | | |
| 1945–46 | 11 | *67* | *9* | *19* | *5* |
| 1947–48 | 43 | | | | |
| 1949–50 | 107 | *67* | *11* | *10* | *11* |
| 1951–52 | 246 | *61* | *19* | *9* | *11* |
| 1953–54 | 351 | *58* | *20* | *11* | *11* |
| 1955–56 | 424 | *66* | *13* | *13* | *8* |
| *Post mortem* | | | | | |
| 1945–46 | 11 | *17* | *23* | *38* | *21* |
| 1947–48 | 36 | | | | |
| 1949–50 | 84 | *20* | *23* | *39* | *18* |
| 1951–52 | 117 | *29* | *23* | *32* | *16* |
| 1953–54 | 163 | *28* | *31* | *26* | *15* |
| 1955–56 | 104 | *18* | *33* | *32* | *18* |
| *Biopsy* | | | | | |
| 1945–46 | 82 | *51* | *18* | *26* | *5* |
| 1947–48 | 175 | *54* | *13* | *31* | *2* |
| 1949–50 | 289 | *53* | *22* | *19* | *6* |
| 1951–52 | 370 | *48* | *23* | *19* | *10* |
| 1953–54 | 379 | *49* | *33* | *17* | *6* |
| 1955–56 | 529 | *43* | *31* | *17* | *9* |

TABLE V.—*Proportions of Oat-cell Tumours to all Anaplastic Tumours (including Oat-cell): Males: Resection only*

| | (Per cent) 1945–50 | 1951–52 | 1953–54 | 1955–56 |
|---|---|---|---|---|
| *Age* | | | | |
| Less than 55 | *59* | *47* | *40* | *58* |
| 55 or more | *50* | *23* | *31* | *41* |

*Oat-cell tumours*

Not all pathologists agree that oat-cell tumours form a group distinct from other anaplastic tumours, and in many published series the two are combined. For this reason our finding that the oat-cell tumour is the only type whose relative frequency is at all strongly associated with age is particularly interesting. In fact Bryson and Spencer (1951) made a similar observation in a series of 866 autopsies; they found the mean age of patients with an oat-cell tumour to be 3 years younger than that of " polygonal cell growths " but they did not comment on the finding.

The age distributions for the 4 types of tumour and three methods of diagnosis are shown in Fig. 1. They show clearly the consistent way in which oat-cell tumours are distinguished from the remainder. The difference was most marked among operation specimens, where the median age was approximately 4 years younger in oat-cell than in the other tumours. This age difference appeared consistently when the material from each centre was analysed separately.

During the period of this survey there was a tendency for the proportion of older patients to increase. The younger patients therefore include relatively more from the earlier years than do the older. If, as is possible, the term " oat-cell " has become less widely used by pathologists over the period, the result would be an apparent association between oat-cell tumours and younger patients. In fact this is not the cause of our finding as may be seen by studying the proportions for shorter periods of time. Table V shows the proportions of oat-cell tumours, calculated from all anaplastic (including oat-cell) tumours, in operation specimens over four such periods.

FIG. 1.—Age distribution of males in 10 year age groups :
Squamous cell carcinoma — — —
Anaplastic carcinoma - - - - -
Oat-cell carcinoma ————

In each period the oat-cell ratio was distinctly higher in younger than in older patients. Calculation of oat-cell ratios for the same periods of time for biopsy and for post mortem diagnosis gave the same results. It is difficult to think of any reason, unrelated to the appearance of the tumours, which would consistently lead pathologists from various parts of the country, and over a period of ten years, to agree in associating the term " oat-cell " with younger patients, regardless of the method of diagnosis. There would seem to be a real distinction between oat-cell and other anaplastic tumours.

*Females*

The smaller number of women does not permit such detailed analysis as is possible for men. A few points do nevertheless emerge from a study of the female

TABLE VI.—*Percentage Frequency of Histological Types : Comparison with Other British Series*

<table>

</table>

In some instances percentages have been recalculated, omitting the unclassified cases.

data. Table I shows that although fewer squamous cell and correspondingly more of each of the other types of tumour were diagnosed among women, there was a pattern similar to that among men for the methods of diagnosis. For example the highest proportion of squamous cell tumours was among resection specimens, while post mortem material contains high proportions of oat-cell tumours and adenocarcinomas.

The figures also show that only 19 per cent of diagnoses among women, compared with 34 per cent among men were made on resected specimens ; post mortem examination accounted for 25 per cent of the diagnoses in women as against 15 per cent for men. These observations might suggest that there were more older women than men in the series. In the series as a whole the women were in fact slightly younger than the men (45 per cent under 55 years of age compared with 39 per cent). The difference was particularly marked in the very young patients as 6 per cent of the women and only 1 per cent of men were under 35 years of age. Of the 25 women under 35 years of age as many as 13 (52 per cent) had oat-cell tumours. Apart from this finding (which may be completely fortuitous) there were no striking variations in the age distributions between the four histological types. There is, however, a suggestion that squamous cell tumours were slightly more common among older women (in spite of the fact that these tumours are found more often at operation) and anaplastic more common among younger patients. A study of time-trends and the distribution according to urbanisation of place of residence yielded nothing of interest.

### DISCUSSION

If a histological diagnosis were available for all cases of lung cancer a complete national registration scheme would provide the ideal basis for the study of the epidemiology of the different histological types ; it would hardly then be necessary to consider the source of the material whether from autopsy, biopsy or resection specimens. Unfortunately it seems that histological data are available for considerably less than half the patients with diagnosed lung cancer in this country. In the early years of Cancer Registration Scheme it was reported that 38 per cent of cases of male lung cancer were proved histologically (Stocks, 1950), but there is reason to suppose that this proportion included cases subjected to no more than a naked-eye examination and that the true figure was, at that time, not much above 30 per cent.

The present series of histologically diagnosed cases probably represents less than a third of all the cases occurring in the South Western Region together with about three quarters of all the cases referred from an indeterminate area to two London teaching hospitals. It cannot therefore be claimed to be free from bias. The large numbers, however, make it possible to allow for some, at least, of the sources of bias by enabling the data to be sub-divided according to several different factors.

A particular difficulty which has been made much of by some writers, for instance Walter and Pryce (1955), is that there is wide variation in criteria of diagnosis among different pathologists. In fact, if we restrict comparisons to this country and make allowances for sex and method of diagnosis the differences between the proportions reported from different centres are remarkably small. This is borne out by the comparisons made within our series and with those of some of the well known British series as shown in Table VI. The Table shows, for example, that there is general agreement that women have fewer squamous cell tumours and more adenocarcinomas than men whatever the method of diagnosis and also that the proportion of undifferentiated tumours is consistently greater in necropsy than in other material.

In spite of their limitations we believe that histological data based on cases registered under the National Cancer Registration Scheme do provide a potentially useful source of information in the epidemiological study of lung cancer. Apart from the bare statement of the proportions of histological types by sex and method of diagnosis our one positive finding has been to confirm that of Bryson and Spencer (1951) that men with oat-cell tumours are, on the average, younger than those with other anaplastic growths. We have already discussed our reasons for believing that this reflects a real difference between the two types of tumour and we would like to echo the plea of Walter and Pryce (1955) that the term " oat-cell "

should be retained and that these tumours should be counted separately in studies of this sort.

Our chief negative result has been that we have been unable to detect any association between cell type and place of residence in whatever way the material was analysed. In view of Kreyburg's (1956) finding of a higher incidence of squamous cell cancer among townsmen than among countrymen it is perhaps suprising that no such differences were found in this material. There is no apparent reason why the selective factors at work in this series should have masked this particular relationship and we only conclude that, if present at all in this country, it is not as well marked as in Norway.

We were able to produce no real evidence, positive or negative, on the question of the changes in the relative proportions of different cell types over the period reviewed. Spain (1959) has recently reviewed the autopsy material in a New York hospital and shown that, relative to adenocarcinoma, squamous and anaplastic growths have increased more than four-fold over a period of forty years.

### SUMMARY

The paper describes a study of the histological diagnosis in 3965 cases of lung cancer registered under the National Cancer Registration Scheme in the South-West of England and in London in the years 1945–56.

The distribution of the main histological types was found to vary considerably according to sex and method of diagnosis (operation, biopsy and post mortem). The findings were similar to those of other workers from this country.

The proportion of " oat-cell " tumours was found to decrease with increasing age (from 30–40 per cent at ages less than 40 to about 15 per cent at ages above 55). This was not true of other anaplastic growths and reasons are given for believing that the finding reflects a real aetiological difference between oat-cell and other anaplastic growths.

No conclusions were drawn from a study of the variations over time in the proportions of the histological types and no relationship was found between the histological type and the area of domicile (town or country).

The advantages and disadvantages of a survey based on the National Cancer registration scheme are discussed.

This survey could not have been undertaken without the active co-operation of many people, but acknowledgment should be made especially to the late Professor Sir Ernest Kennaway under whose guidance it was initiated ; to Mr. R. M. Vick and the staff of the South Western Regional Cancer Records Bureau ; to Dr. W. P. D. Logan and other members of the General Register Office ; to Professor J. W. S. Blacklock, Professor Dorothy Russell and other pathologists at St. Bartholomew's and at the London Hospital ; and to Dr. Richard Doll.

We are also grateful for the help given by the staffs of the records departments in the contributing hospitals and the Department of Medical Statistics at St. Bartholomew's Hospital where the material was analysed on punched cards.

### REFERENCES

BELCHER, J. R.—(1956) *Lancet*, i, 349.
BRYSON, C. L. AND SPENCER, H.—(1951) *Quart. J. Med.* (*new series*), **20**, 173.
DOLL, R. AND HILL, A. B.—(1952) *Brit. med. J.*, ii, 1271.

Galluzzi, S. and Payne, P. M.—(1955) *Brit. J. Cancer*, **9**, 511.
Gifford, J. H. and Waddington, J. K. B.—(1957) *Brit. med. J.*, i, 723.
Kreyberg, L.—(1952) *Brit. J. Cancer*, **6**, 112.—(1954) *Ibid.*, **8**, 199.—(1956) *Brit. J. prev. soc. Med.*, **10**, 145.
Ministry of Health—(1956) Report, Part II, On the State of Public Health, 1955, London (H.M. Stationery Office).
Spain, D. M.—(1959) *J. nat. Cancer Inst.*, **23**, 427.
Stocks, P.—(1950) General Register Office: Studies on Medical and Population Subjects, No. 3—Cancer Registration in England and Wales, London (H.M. Stationery Office).
Walter, J. B. and Pryce D. M.—(1955) *Thorax*, **10**, 107.